# The integrative health status of women with uterine fibroids wishing to maintain fertility: a cross-sectional study based on Levine’s conservation model

**DOI:** 10.4069/whn.2025.03.02

**Published:** 2025-03-28

**Authors:** Hye Moon Kim, ChaeWeon Chung

**Affiliations:** 1 College of Nursing, Seoul National University, Seoul, Korea; 2 Research Institute of Nursing Science, College of Nursing, Seoul National University, Seoul, Korea

**Keywords:** Fertility, Holistic health, Leiomyoma, Uterus, Women

## Abstract

**Purpose:**

The increasing incidence of uterine fibroids (UFs) among women who could consider conception presents challenges. This study examined the relationships among fatigue, UF symptom severity, sexual function, anxiety, and loneliness across diverse life conditions and treatment stages in women with UFs wishing to maintain fertility.

**Methods:**

This descriptive correlational study, guided by Levine’s conservation model, included 221 women with UFs hoping to maintain their reproductive potential. Participants were recruited from gynecology-focused online communities, and data were collected through an online survey. The questionnaire gathered information on general and treatment-related characteristics, as well as measures of fatigue, UF symptom severity, sexual function, anxiety, and loneliness. The data were analyzed using descriptive statistics, independent t-test, one-way analysis of variance, the Welch test, and Pearson correlation analysis.

**Results:**

Most participants (91.9%) experienced fatigue, and sexual dysfunction was prevalent (85.5%). Fatigue, sexual function, anxiety, and loneliness levels were significantly intercorrelated (*p*<.001), whereas UF symptom severity was only associated with fatigue (r=.51, *p*<.001) and sexual function (r=–.41, *p*<.001). Women without specific pregnancy plans exhibited greater anxiety and poorer sexual function than those with plans, and women with low economic status showed poorer outcomes for most variables. Women currently undergoing medical treatment reported the highest UF symptom severity, while those with a longer diagnosis history or not currently receiving treatment exhibited greater anxiety and loneliness.

**Conclusion:**

Women with UFs intending to maintain fertility face multidimensional and interrelated health challenges. Beyond fibroid-focused treatments, clinical nursing and education should integrate physical and psychosocial health indicators while supporting reproductive health.

## Introduction

Uterine fibroids (UFs) are the most prevalent benign neoplasm, affecting approximately 70% of women and significantly impacting their reproductive health [1]. From 1990 to 2019, the number of incident UF cases rose by 67% globally, with the highest risk observed in women aged 35 to 39 [2]. In South Korea, the number of UF patients exceeded 600,000 in 2021, with an annual growth rate of 12.6% over the past 5 years, particularly among women in their 40s and younger [3]. This increase coincides with rising average age of childbirth and growing percentage of mothers of advanced maternal age (35 years and older), which reached 36.3% in 2023 [4]. These demographic changes are expected to lead to a higher incidence of UFs among women who are planning to conceive [5]. Additionally, the adoption of uterus-preserving treatments has significantly increased in recent years [6].

Despite hysterectomy being the only definitive treatment option, the range of treatments available for these women remains limited. Myomectomy has limited long-term data on its effectiveness and carries a risk of recurrence [7]; moreover, uterine artery embolization lacks adequate evidence regarding its impact on future fertility [8]. Most medical treatments target symptoms associated with UFs rather than the fibroids themselves [7], and there are no clear guidelines on whether or when to treat UFs in women who wish to conceive during their reproductive years [5,9]. Women reportedly wait an average of 3.6 years before seeking treatment for UF-related symptoms [10]. Additionally, 25.5% of women with UFs and heavy menstrual bleeding received no treatment within 6 months of diagnosis despite the significant burden of the disease, and 80.5% of those on contraceptive hormone therapy discontinued it [11]. These findings highlight the confusion women face in managing UFs, forcing them to navigate a condition fraught with uncertainty, without clear resolutions for their reproductive health outcomes.

Approximately 20% to 50% of women with UFs experience symptoms such as abnormal uterine bleeding, pelvic pain, or an enlarged uterus [7,12]. Abnormal bleeding is the most common symptom, occurring in 30.6% to 73.3% of diagnosed cases [13]. This symptom often correlates with fatigue, which is the most frequently reported physical impact of UFs, affecting 86.7% of women who experience heavy bleeding [14]. Anemia, found in over half of the UF patients in outpatient gynecology clinics [15], along with reduced physical activity—more commonly reported among younger women (under 40 years-old) [10]—and sleep disturbances due to frequent changes of sanitary products [14], can contribute to fatigue. Additionally, these physical impacts may be linked to negative emotions associated with UFs, including fear, discouragement [16,17], anxiety, anger, depression, and helplessness [16,18]. These emotions persist from before diagnosis and throughout the course of the condition. The uncertainty of the UF disease process can further exacerbate anxiety [19], with affected women exhibiting higher anxiety levels than those in the general population [20,21].

Furthermore, women’s physical and emotional responses to their condition were often linked to a perceived lack of control, particularly affecting their social lives. This resulted in reduced social activities and weakened relationships [16,18]. In large-scale studies, approximately one-third of women consistently reported that UFs adversely affected their job performance [22], attendance at work, and career potential [10]. These challenges were further linked to financial burdens [14,23]. Qualitative research has shown that women with symptomatic UFs found it challenging to maintain their usual lifestyle [14,16], often avoiding outdoor activities, and limiting social interactions, which frequently led to social isolation and feelings of loneliness [18,24]. Most notably, UFs affected relationships with a spouse or partner in over 20% of cases [10,25] and impacted the sexual lives of 42.9% of women [22]. The negative effects on sexual life were complex, affecting women’s sense of femininity or sexuality [10,25], and their romantic relationships [14,16], with dyspareunia reported to be 2.8 times more common [26]. Therefore, the challenges presented by UFs are interconnected and multifaceted, particularly for women who are trying to preserve their fertility while managing societal expectations during their reproductive years.

However, holistic approaches to addressing these challenges are still relatively uncommon. To address these gaps, this study utilized Levine’s conservation model, which views individuals as holistic beings striving to maintain their integrity [27,28]. The concept of conservation encompasses four dimensions—energy, structural integrity, personal integrity, and social integrity. These dimensions function cohesively rather than in isolation, as they are “joined within the individual” [29,30]. The model has served as a basis for integrative care for postpartum [31,32] and infertile women [33,34], proving effective in reducing fatigue and enhancing physical, psychological, and social health outcomes. These findings indicate its potential as a framework for managing health changes associated with reproductive organs, especially in cases involving UFs and fertility issues.

**Figure 1.**
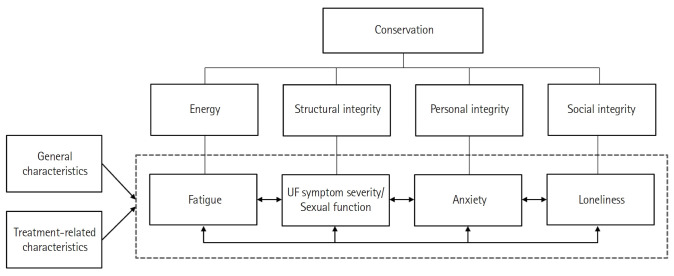
Theoretical framework and substructure of the study, based on Levine’s conservation model. UF: Uterine fibroid.

Fatigue is a key variable that involves an imbalance in energy conservation. Given the challenges inherent in the disease process of UF for women wishing to maintain fertility, anxiety reflects personal integrity as a significant variable. Additionally, sexual function, which is not fully captured by the symptom severity of UF, should be investigated as a variable reflecting structural integrity. For social integrity, we focused on the unintended exclusion of women with UF from work, family, and social activities, and the resulting subjective distress in the form of loneliness. Therefore, this study aimed to investigate the relationships among fatigue, UF symptom severity, sexual function, anxiety, and loneliness, as well as to assess the levels and differences based on general and treatment-related characteristics in women with UFs who wish to maintain fertility (Figure 1). By adopting a holistic perspective, we can gain valuable insights into addressing health issues across these four dimensions, which could lead to targeted strategies and more accessible interventions.

## Methods

**Ethics statement:** This study was approved by the Institutional Review Board of Seoul National University (2310/001-003). Participants were given information on the study and responding to the online survey was considered as consenting to the study.

### Study design

This study utilized a descriptive correlational design and employed a cross-sectional survey. It adhered to the STROBE reporting guidelines (http://www.strobe-statement.org).

### Sample and sampling

The participants in this study were recruited through convenience sampling. Women were eligible for inclusion if they were between the ages of 19 and 49 years, had a clinical diagnosis of UF, wished to maintain their fertility, retained the function of at least one ovary and the uterus, and had engaged in sexual intercourse at least once in the past 4 weeks. The exclusion criteria encompassed women who were currently pregnant, had naturally resolved UFs, or were postmenopausal.

The minimum sample size for this study was calculated using G*Power, resulting in a requirement of 200 participants. This calculation was based on an analysis of variance with a medium effect size of 0.25, a significance level of 0.05, a power of 0.80, and five groups. Anticipating a 25% dropout rate, as observed in a previous online survey study on UFs [22], the target sample size was set at 267. A total of 267 women were recruited and after duplicates and insufficient responses were excluded, data on 221 participants were analyzed.

### Measurements

The survey collected data on fatigue, UF symptom severity, sexual function, anxiety, and loneliness, based on Levine’s conservation model. Permission to use the instruments in this study was obtained prior to data collection.

#### Fatigue

Fatigue was measured using the Korean version [35] of the Fatigue Severity Scale (FSS) [36]. The FSS comprises nine items that assess levels of fatigue experienced over the previous week, each rated on a 7-point Likert scale ranging from 1 (strongly disagree) to 7 (strongly agree). The point-average score is calculated (possible range, 1–7), and higher scores indicate greater fatigue. Chung and Song [35] determined a cutoff score of 3.22 to differentiate between the fatigue and control groups. Cronbach’s α was .94 in the Korean version at development [35] and .93 in this study.

#### Uterine fibroid symptom severity

UF symptom severity was measured using the Symptom Severity Scale (SSS) from The Uterine Fibroid Symptom and Quality of Life (UFS-QoL) questionnaire [37]. The UFS-QoL, which is endorsed by Mapi Research Trust, also includes a Korean version. The SSS evaluates eight symptoms, including heavy menstrual bleeding, pelvic pain, frequent urination, and fatigue, based on the patient’s experiences over the past 3 months. Each symptom is rated using a 5-point Likert scale, ranging from 1 (not at all) to 5 (a very great deal). The scores are then converted to a 100-point scale, where higher scores (possible range, 0–100) reflect greater symptom severity. The Cronbach’s α was .86 at the time of development [37] and .88 in this study.

#### Sexual function

Sexual function was assessed using the Korean version [38] of the Female Sexual Function Index-6 [39] (FSFI-6K). The FSFI-6K comprises six items that evaluate the following domains over the previous 4 weeks: desire, arousal, lubrication, orgasm, satisfaction, and pain. The domains of desire and satisfaction are rated on a scale from 1 to 5, whereas the other domains are rated from 0 to 5. The total score is derived by adding the scores from each domain, with higher scores (possible range, 2–30) indicating better sexual function. The cutoff score for diagnosing female sexual dysfunction was set at ≤21. The Cronbach’s α was .89 in the Korean version at development [38] and .87 in this study.

#### Anxiety

Anxiety was measured using the Korean version [40] of the State-Trait Anxiety Inventory (STAI) [41]. The STAI includes two subscales: state anxiety and trait anxiety, each containing 20 items rated on a 4-point Likert scale (1=not at all/almost never to 4=very much so/almost always). Higher scores (possible range for each subscale, 20–80) indicate greater anxiety. The Cronbach’s α values for state and trait were .89 and .82 in the Korean version at development [40] and .96 and .95, respectively, in this study.

#### Loneliness

Loneliness was assessed using the 20-item Korean version [42] of the UCLA Loneliness Scale version 3 [43]. This scale is rated on a 4-point Likert scale (1=never to 4=always) and higher scores (possible range, 20–80) indicate greater loneliness. The Cronbach’s α was .93 in the Korean version at development [42] and .97 in this study.

#### General and treatment-related characteristics

General characteristics included age, marital status, whether participants had children, plans for pregnancy, employment status, and subjective economic status. Treatment-related characteristics encompassed the duration since UF diagnosis and the history of treatments received.

### Data collection

To ensure a diverse range of responses from women at various stages of treatment and living conditions, data collection was carried out through an online survey from January 30 to February 16, 2024. Permission to post an Institutional Review Board-approved recruitment advertisement was granted by the administrators of four online communities focused on gynecological diseases (e.g., https://cafe.naver.com/mOOOOO, which has about 200,000 members). Interested participants were asked to contact the researchers, who then verified that they met the inclusion and exclusion criteria before providing a URL to the survey site. The survey began with a detailed description of the study, and only those who expressed their consent were permitted to continue. To avoid multiple entries from the same individual, the survey was configured to allow only one submission per IP address. The survey took roughly 10 to 20 minutes to complete, and as a token of appreciation, participants received an online coupon (worth approximately 5 US dollars) upon completion.

### Data analysis

The collected data were analyzed using SPSS Statistics, with statistical significance set at *p*<.05. We assessed the general characteristics, treatment-related characteristics, and measured variables of the participants using descriptive statistics after conducting a normality test based on skewness and kurtosis. Differences in fatigue, UF symptom severity, sexual function, anxiety, and loneliness based on general and treatment-related characteristics were evaluated using independent t-tests and one-way analysis of variance, with subsequent post-hoc analysis conducted via the Scheffé test. In cases of variance heterogeneity, the Welch test was utilized for analysis, followed by post-hoc tests employing the Games-Howell method. The relationships among fatigue, UF symptom severity, sexual function, anxiety, and loneliness were analyzed using Pearson correlation coefficients.

## Results

### General and treatment-related characteristics of the participants

The mean age of the participants was 34.08 (±4.55) years. Of these, 53.4% were married, 78.7% did not have children, 76.5% were employed, and 51.1% described their economic status as ‘average.’ Regarding their plans for pregnancy, 54.8% indicated that they had no specific plans but were open to the possibility in the future. Another 24.4% had specific plans, though not for the immediate future, and 20.8% were actively trying to conceive. In terms of treatment-related characteristics, the average time since UF diagnosis was 2.91 (±1.98) years. Among the participants, 21.7% had undergone a myomectomy. With respect to their experience of medical treatment, 56.1% were observed clinically without any medication, 27.6% had previously received treatment but were not currently on any, and 16.3% were actively receiving medical treatment. Of those who had been medicated, 67.0% had experience with non-hormonal treatments (Table 1).

### Participants’ levels of fatigue, uterine fibroid symptom severity, sexual function, anxiety, and loneliness

Fatigue had a relatively high mean score of 4.93 (±1.06), and 91.9% of participants scored above the cutoff of 3.22, placing them within the fatigue group range. UF symptom severity was at a mid-level, with a score of 46.37 (±20.89) out of 100, similar to the original mean [37]. Sexual function was low, with a mean score of 17.35 (±4.13) and 85.5% of participants scoring ≤21 (cutoff), indicative of female sexual dysfunction. State anxiety and trait anxiety scores were relatively high and closely matched, averaging 47.52 (±14.67) and 47.75 (±13.01) respectively, both displaying wide ranges. Loneliness was at a moderate level, with an average score of 41.79 (±15.22) (Table 1).

### Differences in fatigue, uterine fibroid symptom severity, sexual function, anxiety, and loneliness according to participants’ characteristics

Differences in the main variables by participants’ characteristics are presented in Table 2. Fatigue levels varied significantly based on the time since diagnosis (F=5.54, *p*=.001), experience of medical treatment (F=5.08, *p*=.008), and subjective economic status (F=13.23, *p*<.001). Women diagnosed over 5 years ago exhibited the highest fatigue scores, significantly higher than those diagnosed between one and less than 3 years ago, as indicated by post-hoc analysis. Participants currently undergoing medical treatment reported higher fatigue levels compared to those under clinical observation. Additionally, women with a low subjective economic status experienced greater fatigue compared to those with high or average economic status. UF symptom severity showed significant differences only in relation to the experience of medical treatment (F=27.64, *p*<.001). Those currently receiving treatment reported the highest symptom severity, followed by those who had received treatment in the past and those in the observation group. Sexual function also varied significantly depending on pregnancy plans (F=4.07, *p*=.020). Women with specific plans for pregnancy demonstrated better sexual function compared to those without specific plans. Moreover, women with low subjective economic status exhibited the poorest sexual function.

Meanwhile, state anxiety and trait anxiety exhibited similar trends across participant characteristics, with both significantly higher in women without specific pregnancy plans compared to those actively trying to conceive. Women diagnosed more than 5 years ago had significantly higher anxiety scores than those diagnosed within the last 5 years. Conversely, those undergoing medical treatment had the lowest anxiety scores. Loneliness levels varied according to work status (F=–3.48, *p*=.001), subjective economic status (F=6.77, *p*=.001), time since diagnosis (F=8.66, *p*<.001), history of myomectomy (F=5.10, *p*<.001), and experience of medical treatment (F=4.80, *p*=.010). Women who were currently employed, had a high subjective economic status, were diagnosed within the past year, or were receiving medical treatment reported lower loneliness scores. In contrast, those diagnosed over 5 years ago or with a history of myomectomy reported higher loneliness scores.

Among the general characteristics examined in this study, age, marital status, and presence of children showed no significant relationships with the main study variables.

### Relationships among participants’ fatigue, uterine fibroid symptom severity, sexual function, anxiety, and loneliness

Fatigue (r=.32–.51, *p*<.001) and sexual function (r=–.50 to –.41, *p*<.001) were significantly correlated with all other variables. Significant correlations were also observed among most variables; however, UF symptom severity showed no association with anxiety or loneliness (Table 3).

## Discussion

This study is the first, to our knowledge, to assess the challenges encountered by women with UFs who wish to maintain their fertility, using Levine’s conservation model as a framework. The results indicated that the four dimensions of integrity are significantly interconnected and complex, influenced by both general and treatment-specific characteristics.

Fatigue, the most prevalent physical effect of UFs, mirrors the overall energy levels of the women involved, with 91.9% of participants falling into the fatigued category. The average fatigue score of 4.93 not only surpassed that of the normative control group, which stood at 2.19, but also exceeded the fatigue group’s average of 4.35 [35]. This score is nearly on par with levels seen in patients with chronic fatigue syndrome, who had an average score of 5.04 [44]. The substantial fatigue observed correlates with all other variables studied, suggesting that these women’s broader health challenges stem from an inability to conserve energy, especially those who wish to preserve their reproductive potential for future conception. Furthermore, fatigue likely plays a crucial role in female fertility, affecting hormonal balance, immune function, and cellular energy production [45]. Consequently, addressing fatigue should be a priority in this population.

Studies that applied the conservation model to postpartum women [31,32] and those experiencing infertility [33,34] have shown that energy-generating activities such as yoga and Pilates can effectively reduce fatigue. This suggests that these strategies may also be beneficial for women with UFs. Moreover, optimizing energy use is crucial, given the varied social roles women of reproductive age play, particularly in light of the direct relationship between fatigue, the varying severity of bleeding, and the disruptions it causes in daily life. Systematic examination of daily routines and monitoring of hemoglobin levels can help pinpoint the triggers of fatigue. Understanding the fluctuations in energy levels—possibly in sync with the cyclical nature of their symptoms—enables women to rearrange their activities and prioritize tasks during times of higher energy. This adjustment can also better prepare them for future pregnancy plans. Educational nursing interventions can facilitate this process by empowering women to manage fatigue proactively and tailor strategies to their individual contexts.

Similar to the pattern observed with fatigue, the proportion of women with sexual dysfunction (85.5%) was remarkably higher than that of general Korean women (46.7%) [46] and even higher than that of pregnant women (74.9%) [47]. Likewise, the average sexual function score (17.35) was also lower than that of obstetric and gynecologic outpatients (18.9), with consistently lower scores across all sexual function domains [38]. These findings are particularly concerning because sexual dysfunction has been suggested as a possible factor contributing to the negative impact of UFs on fertility [48]. This underscores the importance of addressing sexual function in women with UFs, especially in the context of their reproductive goals.

While numerous studies have reported improvements in sexual function following specific treatments for UFs [49,50], there remains a scarcity of research focused on managing sexual function in women with an intact uterus who live with UFs. Since better sexual function is linked to open discussions about sexual matters with partners [51] and healthcare professionals [52], it is essential to promote transparent and informed conversations about sexual health. This approach is a vital initial step in addressing sexual function in the care of women with UFs. In this study, variations in sexual function were solely attributed to differences in pregnancy plans. Women with specific pregnancy plans exhibited better sexual function compared to those without such plans. This difference may highlight the opportunities for these women to address sexual issues during their detailed pregnancy planning process. Furthermore, significant correlations between sexual function and all other studied variables indicate that sexual function could be a comprehensive indicator of the broader impacts of UFs on women’s lives. In fact, yoga and Pilates were found to improve sexual function in pregnant women [53] and in premenopausal women with sexual dysfunction [54], respectively. These energy-generating strategies may also be effective in improving sexual function in our study population, where fatigue was a prominent issue. Therefore, the primary nursing strategy should focus on the intricate interplay between UF-related challenges and their potential effects on sexual function. It is crucial to ensure that these aspects are incorporated into discussions with healthcare providers and partners.

Interestingly, while UF symptoms were not noted as severe, the state anxiety score observed in this study (47.52) was close to the levels reported in women diagnosed with serous ovarian cancer (47.96) [55]. This suggests that, despite their benign nature, UFs can be perceived as a significant threat. In research focusing on anxiety among first-time UF patients, the trait anxiety score was recorded at 41.9, with UF-related fears such as “no possibility of pregnancy” and “loss of the uterus” showing a significant correlation with trait anxiety (r=.37, *p*<.001) [20]. In contrast, the current study recorded higher trait anxiety scores (47.75), indicating that the increased and persistent anxiety might be due to uncertainties and concerns about fertility. Notably, women without specific plans for pregnancy reported significantly higher anxiety levels than those who were actively trying to conceive. This difference could be attributed to less frequent discussions about the impact of UFs on fertility, potentially leading to internalized concerns. These findings underscore the importance of tailored counseling and proactive interventions to address fertility-related concerns and alleviate persistent anxiety in this population.

Unlike other measured dimensions, loneliness levels (41.79) remained within normative ranges when compared to college students from the original scale development study (40.08) [43] and those from the Korean validation study (41.46) [42]. The participants in this study were recruited from online communities for individuals with similar conditions, which may have helped conserve their social integrity. Notably, a stronger correlation between trait anxiety and loneliness (r=.82, *p*<.001) was observed in our sample compared to that in the Korean validation study (r= .68, *p*<.001) [41]. Additionally, the larger standard deviations for both anxiety and loneliness scores suggest significant individual variation, highlighting the importance of exploring the factors that contribute to these differences.

Women diagnosed for over 5 years and those with a history of myomectomy reported greater anxiety and loneliness in this study. Since there were no differences in UF symptom severity based on the time since diagnosis or a history of myomectomy, these women likely faced ongoing UF-related challenges. UF recurrence rates exceed 30% within 3 years and 50% within 5 years post-myomectomy [56], compared to lower rates (17.1%) among premenopausal women over 45 [57]. This suggests that women of childbearing age in our study, who are seeking to maintain their fertility, encounter a higher risk of recurrence and a prolonged disease burden, which contributes to increased anxiety and loneliness. Therefore, it is crucial to prioritize nursing interventions that address the psychological impact and provide targeted support for women dealing with long-term and recurring UFs.

Another noteworthy finding was the variation in key variables based on the experience of medical treatment. Women currently undergoing medical treatment reported the highest UF symptom severity, indicating that they are the most affected by these symptoms. Importantly, the study participants were not only managing these symptoms but were also dealing with concerns about fertility while seeking relevant information. Consequently, they exhibited the highest levels of fatigue but the lowest levels of anxiety and loneliness among all groups. This could be attributed to the comforting effects of regular medical interactions. This comforting effect might also explain why, contrary to expectations, no significant correlations were found between UF symptom severity and levels of anxiety or loneliness.

Accordingly, the primary concern may lie with women who are overlooked by medical attention or who do not pursue follow-up care. This is where nursing interventions, leveraging online platforms, can make a significant impact. In our study, 83.7% of participants were not currently undergoing medical treatment. Although this study did not examine the specific purposes for which participants used these communities, such platforms have the potential to provide reassurance through information-seeking and to bridge gaps in insufficient in-person social networks. Given the sensitive nature of UFs and fertility, these platforms can also offer a private and supportive space for women to seek information and address their concerns. The internet is a key information source for women with UFs [20], but they express a preference for high-quality information from healthcare professionals [58]. In this context, utilizing online platforms to deliver reliable, accessible educational interventions addressing symptom management and fertility concerns could serve as an effective medium for meeting these needs.

Regarding the women’s general characteristics, low subjective economic status was associated with worse physical, psychological, and social health indicators among women with UFs wishing to maintain fertility. Despite experiencing similar levels of UF symptom severity, women of lower economic status may suffer greater fatigue, anxiety, loneliness, and diminished sexual health due to additional barriers. These barriers encompass the costs associated with hygiene products, diagnostic tests, and fertility treatments, potentially delaying prevention, screening, and treatment efforts [59]. Therefore, it is crucial to recognize the social determinants impacting this group to ensure that vulnerable populations are adequately addressed within nursing care practices.

A limitation of this study is that it recruited participants from online communities focused on gynecological diseases. This approach may have resulted in the inclusion of individuals specifically motivated to seek information on UFs or discuss sexual health, potentially introducing selection bias and limiting the generalizability of the results to broader populations. Furthermore, since all data on UFs were self-reported and not verified against medical records, the study may be prone to recall bias. Despite these limitations, this study is unique in its focus on women who wish to maintain fertility and in its recruitment of participants from non-medical settings, which allowed for a diverse range of backgrounds. Given the variability in UF manifestations, symptoms, and treatment options, this research enhances our understanding of the less explored aspects of UFs in women.

In conclusion, this study sheds light on the multifaceted challenges faced by women with UFs who wish to maintain their fertility. It emphasizes the interconnected physical, psychological, and social dimensions they encounter, including fatigue, symptom severity, sexual function, anxiety, and loneliness. The significant fatigue observed, comparable to that seen in chronic conditions, underscores the critical need for prioritizing fatigue management to help conserve energy. Additionally, addressing fertility concerns is crucial for effectively managing anxiety. Given the strong association between loneliness and anxiety, nursing interventions that bridge gaps in consistent medical engagement—especially for those with long-standing or recurrent cases—represent an impactful strategy. Furthermore, interventions that address the interplay of these factors could improve sexual function, a vital aspect of women’s quality of life. Collectively, these strategies can help women better adapt to the challenges of UFs while supporting their integrative health and reproductive well-being.

## Figures and Tables

**Table 1.** Participants’ characteristics and descriptive statistics of the measured variables (N=221)

Variable	Categories	n (%)	Mean ±SD	Data range
Age (year)	19–30	31 (14.0)	34.08±4.55	23–45
	30–34	86 (38.9)		
	35–39	79 (35.7)		
	40–49	25 (11.3)		
Marital status	Married	118 (53.4)		
	Never married	100 (45.2)		
	Separated/divorced	3 (1.4)		
Having child(ren)	Yes	47 (21.3)		
	No	174 (78.7)		
Pregnancy plan	Actively trying	46 (20.8)		
	Specific plan	54 (24.4)		
	No specific plan	121 (54.8)		
Employment status	Employed	169 (76.5)		
	Unemployed	52 (23.5)		
	Housewife	30 (13.6)		
	Leave of absence	11 (5.0)		
	Looking for a job	7 (3.2)		
	Student	4 (1.8)		
Subjective economic status	High	86 (38.9)		
	Average	113 (51.1)		
	Low	22 (10.0)		
Time since diagnosis (yr)	<1	42 (19.0)	2.91±1.98	0.17–9.58
	1–2.9	88 (39.8)		
	3–4.9	53 (24.0)		
	≥5	38 (17.2)		
History of myomectomy	Yes	48 (21.7)		
	No	173 (78.3)		
Experience of medical treatment	Current	36 (16.3)		
	Past	61 (27.6)		
	None	124 (56.1)		
Types of medical treatment^†^ (n=97)^‡^	Non-hormonal	65 (67.0)		
	Oral hormonal	30 (30.9)		
	Shot hormonal	26 (26.8)		
	LNG-IUS	6 (6.2)		
Fatigue	<3.22	18 (8.1)	4.93±1.06	1.89–6.67
	≥3.22^§^	203 (91.9)		
UF symptom severity			46.37±20.89	0–90.63
Sexual function	≤21^§^	189 (85.5)	17.35±4.13	6–27
	>21	32 (14.5)		
State anxiety			47.52±14.67	20–76
Trait anxiety			47.75±13.01	25–76
Loneliness			41.79±15.22	20–76

LNG-IUS: Levonorgestrel-releasing intrauterine system; UF: uterine fibroid.

† Non-hormonal: e.g., nonsteroid anti-inflammatory drugs, tranexamic acid; oral hormonal: e.g., combined oral contraceptive, progestogen; shot hormonal: e.g., gonadotropin-releasing hormone analog.

‡ Multiple response.

§ Cutoff score.

**Table 2.** Differences in fatigue, UF symptom severity, sexual function, anxiety, and loneliness according to the participant’s characteristics (N=221)

Characteristic	Categories	Fatigue		UF symptom severity		Sexual function		State anxiety		Trait anxiety		Loneliness	
		Mean±SD	t/F(*p*) Scheffé	Mean±SD	t/F(*p*) Scheffé	Mean±SD	t/F(*p*) Scheffé	Mean±SD	t/F(*p*) Scheffé	Mean±SD	t/F(*p*) Scheffé	Mean±SD	t/F(*p*) Scheffé
Age (yr)	<35 (n=117)	4.87±1.03	–0.86 (.392)	46.50±20.40	0.10 (.919)	17.09±4.22	–1.00 (.317)	48.12±15.63	0.64 (.524)	48.38±14.00	0.77 (.441)	42.08±16.81	0.30 (.762)
	≥35 (n=104)	5.00±1.10		46.21±21.52		17.64±4.02		46.86±13.54		47.03±11.83		41.46±13.26	
Spouse presence	Yes (n=118)	5.01±1.16	1.20 (.230)	47.35±23.40	0.76 (.445)	17.27±4.52	–0.30 (.764)	47.67±14.89	0.16 (.876)	47.69±13.09	–0.73 (.942)	42.28±14.19	0.51 (.608)
	No (n=103)	4.84±0.92		45.24±17.62		17.44±3.65		47.36±14.47		47.82±12.99		41.22±16.36	
Having child(ren)	Yes	5.03±1.15	0.71 (.481)	43.95±21.52	–0.89 (.373)	17.47±3.76	0.22 (.823)	45.81±13.78	–0.90 (.367)	47.00±11.55	–0.44 (.659)	42.83±13.51	0.53 (.598)
	No	4.90±10.3		47.02±20.73		17.32±4.23		47.99±14.90		47.95±13.41		41.51±15.69	
Pregnancy plan	Actively trying^a^	4.84±1.33	0.93 (.398)^†^	49.93±26.87	0.73 (.486)^†^	17.28±5.32	4.07 (.020)^†^ b>c	43.13±14.95	3.06 (.049)a<c	43.57±13.83	4.40 (.013) a<c	39.11±14.47	2.67 (.074)^†^
	Specific plan^b^	4.80±1.13		44.04±20.74		18.56±3.65		47.20±14.40		46.50±11.82		39.39±12.03	
	No specific plan^c^	5.02±0.90		46.05±18.21		16.83±3.72		49.34±14.43		49.89±12.85		43.88±16.50	
Employment status	Employed	4.96±1.06	0.64 (.522)	46.84±21.20	0.60 (.546)	17.45±4.19	0.66 (.512)	46.98±15.26	–1.10 (.273)	47.01±13.35	–1.52 (.131)	39.86±15.20	–3.48 (.001)
	Unemployed	4.85±1.05		44.83±19.96		17.02±3.97		49.29±12.50		50.13±11.64		48.06±13.61	
Subjective economic status	High^a^	4.75±1.25	13.23 (<.001)^†^ a,b<c	44.66±21.21	1.09 (.338)	18.34±4.42	8.5 (<.001)^†^ a,b>c	44.67±15.54	2.91 (.057)	44.44±13.69	5.15 (.007) a<b,c	37.81±15.85	6.77 (.001) a<b,c
	Average^b^	4.96±0.95		46.57±21.60		16.98±3.98		48.99±14.15		49.40±12.40		43.27±14.21	
	Low^c^	5.49±0.43		51.99±14.70		15.36±2.52		51.14±12.25		52.18±10.75		49.68±13.78	
Time since diagnosis (yr)	<1^a^	4.97±0.96	5.54 (.001)^†^ b<d	44.05±20.18	0.49 (.693)^†^	18.21±4.65	1.42 (.241)^†^	48.90±12.96	11.52 (<.001)^†^ a,b,c<d	48.29±12.62	11.79 (<.001)^†^ a,b,c<d	39.11±14.42	8.66 (<.001)^†^ a<c,d b<d
	1~2.9^b^	4.66±1.15		46.20±23.86		17.50±4.34		43.15±15.29		44.40±12.96		39.39±15.03	
	3~4.9^c^	5.09±1.16		46.76±20.01		16.92±4.14		47.81±15.75		47.75±14.71		43.88±16.47	
	≥5^d^	5.29±0.57		48.77±15.13		16.63±2.70		55.74±8.79		54.89±17.23		43.88±10.90	
History of myomectomy	Yes	5.04±0.86	0.98 (.329)	45.83±18.91	–0.20 (.842)	17.27±3.27	–0.17 (.864)	53.85±10.20	4.32 (<.001)	52.50±9.13	3.62 (<.001)	49.48±10.51	5.10 (<.001)
	No	4.90±1.11		46.51±21.45		17.37±4.35		45.77±15.24		46.43±13.63		39.65±15.65	
Experience of medical treatment	Current^a^	5.28±0.88	5.08 (.008)^†^ a>c	63.89±17.12	27.64 (<.001)^†^ a>b>c	16.56±3.55	1.27 (.284)^†^	40.14±11.31	11.15 (<.001)^†^ a<b,c	43.19±10.31	7.06 (.001)^†^ a<b	38.17±11.09	4.80 (.010)^†^ a<b
	Past^b^	5.09±0.82		50.87±14.32		17.11±2.78		51.87±12.60		51.48±10.99		45.70±13.05	
	None^c^	4.75±1.17		39.06±21.05		17.69±4.78		47.53±15.67		47.23±14.16		40.91±16.85	

UF: Uterine fibroid.

† Heteroskedasticity.

**Table 3.** Correlations among study variables (N=221)

Variable	r (*p*)				
	Fatigue	UF symptom severity	Sexual function	State anxiety	Trait anxiety
Fatigue	1				
UF symptom severity	.51 (<.001)	1			
Sexual function	–.49 (<.001)	–.41 (<.001)	1		
State anxiety	.40 (<.001)	.04 (.574)	–.46 (<.001)	1	
Trait anxiety	.43 (<.001)	.06 (.366)	–.50 (<.001)	.93 (<.001)	1
Loneliness	.32 (<.001)	.05 (.462)	–.49 (.001)	.75 (<.001)	.82 (<.001)

UF: Uterine fibroid.
